# Psychological distress, suicidality and resilience of Lithuanian nurses

**DOI:** 10.1186/s12912-024-02632-2

**Published:** 2024-12-18

**Authors:** Povilas Kavaliauskas, Evaldas Kazlauskas, Giedre Smailyte

**Affiliations:** 1 Department of Public Health, Institute of Health Sciences, Faculty of Medicine, Vilnius University, M. K. Čiurlionio Str. 21/27, Vilnius, LT-03101 Lithuania; 2 Laboratory of Cancer Epidemiology, National Cancer Institute, Vilnius, Lithuania; 3 Center for Psychotraumatology, Institute of Psychology, Vilnius University, Vilnius, Lithuania

**Keywords:** Mental health, Depression, Lithuania, Nurses, Suicide risk

## Abstract

**Background:**

Nurses, like other healthcare workers, are prone to poorer mental health, increased burnout, and may have an increased risk of suicide.

**Purpose:**

This study aimed to evaluate mental health problems among Lithuanian nurses and explore factors associated with them.

**Method:**

The survey was completed by 533 nurses. Mental health was assessed using the Depression, Anxiety, and Stress Scale—21, and suicidal ideation was measured with the Suicidal Behaviours Questionnaire—Revised (SBQ-R).

**Findings:**

A large proportion of nurses in the study had high psychological distress, with 18% having high depression, 29.3% - high anxiety, and 17.1% - high stress. 21.2% of the sample had an increased suicide risk. 64.9% of nurses considered changing their careers to a non-medical profession in the last 12 months.

**Discussion:**

Addressing mental health issues in the national healthcare system is critical to avoiding the loss of valued medical community members and ensuring that patients do not lose their critical caretakers.

## Background

Nursing specialists are vital in providing healthcare services [1]. Even though physicians usually lead the medical care team, nurses are leading figures in caring for the patient, being the first person, the patient interacts with during healthcare provision. In recent years, more tasks have been shifted to the nurses [2]. Even though those additional responsibilities are to cope with the shortage of doctors and to provide better care to the patients [2], it can also contribute to staff burnout [3]. Furthermore, workers whose job essence is frequent and intensive interactions with others are at higher risk of experiencing emotional exhaustion, lack of interest in work, problems with interpersonal communication and deteriorating physical health [4].

Studies in various countries reveal challenging work conditions and poor mental health among nursing staff. A national survey in Canada identified that depression was significantly more prevalent among nurses than among other professionals [5]. The prevalence of depression among nurses varies from 25.1 to 35.8% [6, 7]. Nurses are frontline workers with a high workload and often work every day of the week for prolonged hours. However, night shifts negatively affect mental health, causing more frequent depression and worsening of circadian and sleep [8]. Burnout is a common problem among healthcare workers. A study in the U.S. showed that the main reason for nurses leaving their work is burnout [9]. In addition, staff reported a stressful environment and inadequate staffing [9] as crucial factors for leaving their work. The profile of a nurse who is at risk for burnout is a single person with multiple jobs, high workload, and low work experience [3]. However, all nursing specialists can be exposed to burnout, and it does not depend on the position held [10]. Lastly, resilience is important in coping with work challenges and life stressors, as resilience is the process and outcome of successful adaptation to difficult situations through mental, emotional or behavioural flexibility [11]. Resilience may be viewed as a personality trait or feature. However, it may also be conceptualized as a skill which can be developed. A Cochrane meta-analysis demonstrated that resilience training improved resilience, lowered depression levels, and reduced stress levels or stress perception [4].

In Lithuania, there are more than 25,000 nurses [12]. However, according to the experts, there is a 16% lack of nursing specialists in the country. A study from 2009 showed that 23% of nurses had mental distress, with low social support being the most important risk factor [13]. However, the study was conducted 15 years ago, and responders were from a single district community, so they do not reflect the whole of Lithuania. Another previous study showed that 12.9% of nurses experienced bullying in their workplace, but this was conducted in only part of the country [14]. To our knowledge, no other published peer-reviewed articles have analysed this issue. There is a serious lack of well-designed and well-conducted studies that adequately reflect the current mental health situation of Lithuanian nurses. Due to the lack of empirical data on Lithuanian nurses’ mental health, the current study aimed to evaluate mental health, more specifically, anxiety, depression, suicidality, and factors associated with mental health among the nursing specialists in Lithuania.

## Methods

### Participants and procedure

The current study was a part of a broader project of the national healthcare workers’ mental health evaluation in Lithuania. The data was collected from December 2021 to January 2022 using the online survey platform. The invitation to participate in the study was distributed through various professional unions and associations, internal hospital networks, and Lithuanian healthcare workers’ social networks throughout the country. All national and regional healthcare professional organizations and professional unions registered in Lithuania and whose contact information can be acquired were invited to participate. In addition, social network groups of healthcare workers were asked to share invitations to participate in the study for a more comprehensive reach of the medical community in Lithuania nationally. Two additional reminders were sent to healthcare workers to participate in the survey after the release of the initial invitation. In total, 2354 responders opened the online survey, and 1653 completed the questionnaire. After excluding non-medical personnel, the total sample of 1618 licenced healthcare workers finished the survey and met the inclusion criteria for the study. Data from 533 nurses extracted from the larger study working across various regions of Lithuania were included in the study.

### Sociodemographic data and work-related stressors

Sociodemographic data was collected, including gender, age, relationship status (being in a long-term relationship or single), type of work (working in an inpatient, outpatient setting, rehabilitation, palliative care or nursing homes, emergency department or intensive care unit), level of medical service provision (primary, secondary, tertiary), primary work location (size of the city, where responder work), workload and work experience after completion of training. Participants were screened for factors negatively affecting healthcare workers’ mental health extracted from the previously published meta-analyses [15–19]. These factors included poor working conditions, high workload, work with patients, lack of professional development, lack of career perspectives, manager behaviours, mobbing (bullying in the workplace), and exhaustion. Additionally, responders were asked what positive factors are in their workplace: salary (adequate salary to support your life), satisfaction with work, professional development, patients’ gratitude, and support from colleagues. Respondents were asked to indicate if any of those factors affected their daily lives by responding with a binary “yes/no” answer to the items listing these factors.

### Psychological distress and resilience

The Depression, Anxiety and Stress Scale − 21 (DASS-21) was used for the assessment of depression and anxiety [20]. The DASS-21 is a widely used self-report measure that includes three subscales, measuring emotional states of depression, anxiety and stress levels. Each subscale consists of seven items measured on a 4-point Likert scale ranging from 0 (“did not apply to me at all”) to 3 (“applied to me most of the time”). Each DASS-21 subscale provides a score, a sum of responses to each subscale question, with a higher score indicating higher levels of depression, anxiety or stress. The severity of each component was graded by its score: depression (normal/mild < 7; moderate 7–10; severe > 11), anxiety (normal/mild < 6; moderate 6–7; severe > 8), stress (normal/mild < 10; moderate 10–12; severe > 13). The Cronbach alpha for depression, anxiety and stress scales were 0.88, 0.80 and 0,85, respectively.

The Resilience Scale 11 (RS-11) [21] was used to measure psychological resilience. The RS-11 is a unidimensional measure containing 11 items. Each RS-11 item was rated on a 7-point Likert scale ranging from 1 (“do not agree”) to 7 (“agree”). The total RS-11 score ranges from 11 to 77, with a higher score indicating a higher level of resilience. The Cronbach alpha for the RS-11 scale was 0.85.

The Suicidal Behaviors Questionnaire-Revised (SBQ-R) was used to evaluate suicidality in the sample [22]. The SBQ-R comprises four items, each covering a different dimension of suicidality: lifetime suicidal ideation and attempts, the frequency of suicidal ideation over the preceding 12 months, the threat of suicide attempts, and self-reported probability of suicidal behaviour in the future. The four SBQ-R items are rated on Likert scales of varying lengths, resulting in a total score between 3 and 18. Each of them is evaluated with a different number of points. A sum of points of the SBQ-R with a cut-off of ≥ 7 indicates an increased risk for suicide for general population studies. The Cronbach alpha for this scale was 0.81.

### Data analysis

Statistical analysis was performed using IBM SPSS 26.0. A one-way ANOVA test was used to evaluate the association between resilience and psychological distress. Chi-square and Student-t tests were used for univariate analysis to identify statistically significant risk factors for suicidal ideation. Multivariate binary logistic regression was used to assess risk factors for high suicide risk. A dependent variable was a binary variable of high suicide risk (SBQ-R score ≥ 7). Normal values were used as a reference for assessing the role of depression and anxiety in the analysis. Results were held statistically significant when *p* < 0.05.

## Results

### Characteristics of the sample

A total of 533 nurses participated in the study, with a mean age of 43.13 years and an age range of 20 to 69 years. Work experience ranged from 1 to 46 years, with a mean work experience of 20.17 years. In the past 12 months, 64.9% (346) of nurses considered switching to a non-medical profession. The sample was predominantly female, comprising 97.7% of the sample. Table 1 presents the detailed descriptive characteristics of the study sample. The study identified exhaustion and high workload as the primary negative factors, and patients’ gratitude and colleagues’ support as the primary positive factors associated with the nursing job. Table 2 represents the detailed negative and positive factors associated with work.

**Table 1:** Sociodemographic characteristics of the sample (*N* = 533)

Variable	Prevalence (%)
Gender	
Male Female	12 (2.3) 521 (97.7)
Age *M* (*SD*)	43.13 (12.32)
Relationships	
Not in a long-term relationship In a long-term relationship	112 (21) 421 (79)
Having kids	
No kids Having kids	171 (32.1) 362 (67.9)
Type of work	
Outpatient Inpatient Rehabilitation Palliative care or nursing homes Emergency department Intensive care unit	199 (37.3) 246 (46.2) 15 (2.8) 119 (22.3) 90 (16.9) 60 (11.3)
Level of medical service provision	
Primary Secondary Tertiary	212 (39.8) 223 (41.8) 184 (34.5)
Primary workplace location	
One of the five biggest cities Another smaller city Township/rural area	377 (70.7) 125 (23.5) 31 (5.8)
Workload (Full-Time equivalent)	
< 1 FTE 1 FTE > 1 FTE	22 (4.1) 269 (50.5) 242 (45.4)
Average work experience after finished training (years) *M* (*SD*)	20.17 (13.23)

**Table 2:** Negative and positive factors related to the work among nurses (*N* = 533)

Variable	Prevalence (%)
Negative factors	
Poor working conditions	209 (39.2)
High workload	322 (60.4)
Work with patients	124 (23.3)
Lack of professional development	78 (14.6)
Lack of career perspectives	118 (22.1)
Managers	214 (40.2)
Mobbing	193 (36.2)
Exhaustion	356 (66.8)
Positive factors	
Adequate salary	163 (30.6)
Satisfaction with work	258 (48.4)
Professional development	134 (25.1)
Patients’ gratitude	335 (62.9)
Support from colleagues	305 (57.2)

### Resilience and mental health

The average resilience score in the sample was 58.68 (SD ± 9.89), ranging from 22 to 77. Statistically significantly, nurses with high suicide risk had the lower RS-11 scores, which were 59.98 (± 9.52) for the population without suicide risk and 53.85 (± 9.75) for the population with high suicide risk. Spearman’s non-parametric correlation showed a low positive correlation between age and RS-11 score of 0.24 (*p* < 0.001). A positive correlation was found between work experience and the RS-11 score of 0.22 (*p* < 0.001). A one-way ANOVA test was applied to compare resilience across the subsamples having various levels of anxiety and depression levels identified using the DASS-21. The analysis indicated that higher severity of depression and anxiety was significantly associated with lower levels of resilience at *p* < 0.001 (see Table 3).

**Table 3:** Resilience in various levels of depression, anxiety, and stress in the sample

Variable	Prevalence (%)	Resilience M(SD)
Depression		
Normal	181 (34)	63.12 (7.75)
Mild	97 (18.2)	60.43 (9.13)
Moderate Severe	159 (29.8) 62 (11.6)	56.41 (9.45) 52.22 (10.75)
Extremely severe	34 (6.4)	52.41 (10.2)
Anxiety		
Normal	155 (29.1)	62.83 (8.84)
Mild	47 (8.8)	60.29 (10.54)
Moderate Severe	175 (32.8) 72 (13.5)	58.38 (8.61) 56.12 (9.51)
Extremely severe	84 (15.8)	52.92 (10.7)
Stress		
Normal	226 (42.4)	62.93 (8.27)
Mild	101 (18.9)	58.09 (9.77)
Moderate Severe	115 (21.6) 76 (14.3)	54.74 (9.67) 55 (9.13)
Extremely severe	15 (2.8)	47.4 (9.81)

### Factors associated with psychological distress

In the sample, 62 (11.6%) and 34 (3.4%) nurses were identified as having severe and extremely severe levels of depression, respectively. Additionally, 72 (13.5%) and 84 (15.8%) had severe and extremely severe anxiety, and 76 (14.3%) and 15 (2.8%) severe and extremely severe stress, respectively. Univariate analysis for severe and extremely severe depression and anxiety (see Table 4) showed that work in an Outpatient setting was associated with higher levels of depression, and work in an Inpatient was associated with higher levels of anxiety. Career change ideation, poor working conditions, lack of career perspectives, managers, mobbing and exhaustion were associated with high levels of depression among nurses. However, satisfaction with work, professional development and support from colleagues were protective factors associated with lower depression rates (see Table 4). Career change ideation, poor working conditions, managers, mobbing, exhaustion, high workload and working with patients were associated with more frequent anxiety disorders. Meanwhile, satisfaction with your work was associated with lower anxiety levels (see Table 4).

**Table 4:** Variables associated with severe and extremely severe depression and anxiety

Variable	Depression (%)		*p*	Anxiety (%)		*p*
	Normal and moderate	Extreme and extremely severe		Normal and moderate	Extreme and extremely severe	
Gender						
Male Female	12 (100%) 425 (81.6%)	0 (0%) 96 (18.4%)	0.101	11 (91.7%) 366 (70.2%)	1 (8.3%) 155 (29.8%)	0.107
Age *M* (*SD*) ^a^	43.6 (± 12.26)	40.94 (± 12.37)	0.054	43.69 (± 12.26)	41.77 (± 12.36)	0.102
Relationships						
Not in a long-term relationship In a long-term relationship	348 (82.7%) 89 (79.5%)	73 (17.3%) 23 (20.5%)	0.434	302 (71.7%) 75 (67%)	119 (28.3%) 37 (33%)	0.324
Having kids						
No kids Having kids	136 (79.5%) 301 (83.1%)	35 (20.5%) 61 (16.9%)	0.312	113 (66.1%) 264 (72.9%)	58 (33.9%) 98 (27.1%)	0.105
Type of work ^b^						
Outpatient Inpatient Rehabilitation Palliative care or nursing homes Emergency department Intensive care unit	167 (83.9%) 189 (76.8%) 15 (100%) 100 (84%) 72 (80%) 45 (75%)	32 (16.1%) 57 (23.2%) 0 (0%) 19 (16%) 18 (20%) 15 (25%)	0.373 **0.004** 0.081 0.510 0.591 0.135	130 (65.3%) 174 (70.7%) 100 (84%) 85 (71.4%) 62 (68.9%) 44 (73.3%)	69 (34.7%) 72 (29.3%) 19 (16%) 34 (28.6%) 28 (31.1%) 16 (26.7%)	**0.034** 1 0.517 0.855 0.674 0.638
Level of medical service provision*						
Primary Secondary Tertiary	179 (84.4%) 178 (79.8%) 149 (81%)	33 (15.6%) 45 (20.2%) 35 (19%)	0.233 0.269 0.659	142 (67%) 156 (70%) 138 (75%)	70 (33%) 67 (30%) 46 (25%)	0.122 0.738 0.116
Primary workplace location						
One of the five biggest cities Another smaller city Township/rural area	305 (80.9%) 107 (85.6%) 25 (80.6%)	72 (19.1%) 18 (14.4%) 6 (19.4%)	0.486	261 (69.2%) 93 (74.4%) 23 (74.2%)	116 (30.8%) 32 (25.6%) 8 (25.8%)	0.496
Workload (Full-Time equivalent)						
< 1 FTE 1 FTE > 1 FTE	21 (95.5%) 216 (80.3%) 200 (82.6%)	1 (4.5%) 53 (19.7%) 42 (17.4%)	0.193	18 (81.8%) 178 (66.2%) 181 (74.8%)	4 (18.2%) 91 (33.8%) 61 (25.2%)	0.051
Average work experience after finished training (years) *M* (*SD*) ^a^	20.66 (± 13.21)	17.98 (± 13.32)	0.074	20.79 (± 13.11)	18.68 (± 13.52)	0.100
Career change ideation						
Yes No	259 (74.9%) 178 (95.2%)	87 (25.1%) 9 (4.8%)	**< 0.001**	217 (62.7%) 160 (85.6%)	129 (37.3%) 27 (14.4%)	**< 0.001**
Poor working conditions						
Yes No	148 (70.8%) 289 (89.2%)	61 (29.2%) 35 (10.8%)	**< 0.001**	127 (60.8%) 250 (77.2%)	82 (39.2%) 74 (22.8%)	**< 0.001**
High workload						
Yes No	257 (79.8%) 180 (85.3%)	65 (20.2%) 31 (14.7%)	0.106	211 (65.5%) 166 (78.7%)	111 (34.5%) 45 (21.3%)	**0.001**
Work with patients						
Yes No	97 (78.2%) 340 (83.1%)	27 (21.8%) 69 (16.9%)	0.213	77 (62.1%) 300 (73.7%)	47 (37.9%) 109 (26.7%)	**0.016**
Lack of professional development						
Yes No	60 (76.9%) 377 (82.9%)	18 (23.1%) 78 (17.1%)	0.208	50 (64.1%) 327 (71.9%)	28 (35.9%) 128 (28.1%)	0.164
Lack of career perspectives						
Yes No	83 (70.3%) 354 (85.3%)	35 (29.7%) 61 (14.7%)	**< 0.001**	78 (66.1%) 299 (72%)	40 (33.9%) 116 (28%)	0.213
Managers						
Yes No	161 (75.2%) 264 (87.7%)	53 (24.8%) 37 (12.3%)	**< 0.001**	137 (64%) 227 (75.4%)	77 (36%) 74 (24.6%)	**0.005**
Mobbing						
Yes No	141 (73.1%) 296 (87.1%)	52 (26.9%) 44 (12.9%)	**< 0.001**	117 (60.6%) 260 (76.5%)	76 (39.4%) 80 (23.5%)	**< 0.001**
Exhaustion						
Yes No	275 (77.2%) 149 (90.9%)	81 (22.8%) 15 (9.1%)	**< 0.001**	232 (65.2%) 133 (81.1%)	124 (34.8%) 31 (18.9%)	**< 0.001**
Adequate salary						
Yes No	136 (83.4%) 301 (81.4%)	27 (16.6%) 69 (18.6%)	0.564	112 (68.7%) 265 (71.6%)	51 (31.3%) 105 (28.4%)	0.496
Satisfaction with work						
Yes No	222 (86%) 215 (78.2%)	36 (14%) 60 (21.8%)	**0.018**	198 (76.7%) 179 (65.1%)	60 (23.3%) 96 (64.9%)	**0.003**
Professional development						
Yes No	121 (90.3%) 316 (79.2%)	13 (9.7%) 83 (20.8%)	**0.004**	100 (74.6%) 277 (69.4%)	34 (25.4%) 122 30.6%)	0.252
Patients’ gratitude						
Yes No	279 (83.3%) 158 (79.8%)	56 (16.7%) 40 (20.2%)	0.312	231 (69%) 146 (73.7%)	104 (31%) 52 (26.3%)	0.241
Support from colleagues						
Yes No	259 (84.9%) 178 (78.1%)	46 (15.1%) 50 (21.9%)	**0.042**	224 (73.4%) 153 (67.1%)	81 (26.6%) 75 (32.9%)	0.112

Note. Chi-Square was used for all univariate tests, except if indicated - ^a^ Student t-test was used as a statistical model for comparison

^b^ responders could choose more than one response option

### Suicidality among the nurse population

In the sample, 113 nurses (21.2%) scored ≥ 7 on the SBQ-R questionnaire, indicating an increased risk for suicide. Additionally, 27 nurses (5.1%) reported having a suicide plan, and 8 nurses (1.5%) reported previous suicide attempts. Univariate analysis showed that having no children, ideation to change work, poor working conditions, lack of professional development, lack of career perspectives, managers, exhaustion and low satisfaction with work were significantly associated with high suicide risk. Table 5 presents the detailed results of the univariate analysis of the factors related to suicidality.

**Table 5:** Univariate analysis of factors associated with suicidality

Variable	Suicide risk		*p*
	Low suicide risk (SBQ-*R* < 7 ) (*N* = 412)	High suicide risk (SBQ-*R* ≥ 7) (*N* = 113)	
Gender			
Male Female	8 (66.7%) 412 (719%)	4 (33.3%) 109 (20.9%)	0.298
Age *M* (*SD*) ^a^	43.64 (± 12.28)	41.22 (± 12.31)	0.064
Relationships			
Not in a long-term relationship In a long-term relationship	326 (82.7%) 94 (83.9%)	95 (22.6%) 18 (16.1%)	0.135
Having kids			
No kids Having kids	126 (73.7%) 294 (81.2%)	45 (26.3%) 68 (18.8%)	**0.047**
Type of work ^b^			
Outpatient Inpatient Rehabilitation Palliative care or nursing homes Emergency department Intensive care unit	157 (78.9%) 189 (76.8%) 14 (93.3%) 92 (77.3%) 72 (80%) 50 (83.3%)	42 (21.1%) 57 (23.2%) 1 (6.7%) 27 (22.7%) 18 (20%) 10 (16.7%)	0.973 0.324 0.162 0.650 0.764 0.362
Level of medical service provision*			
Primary Secondary Tertiary	168 (79.2%) 175 (78.5.8%) 142 (77.2%)	44 (20.8%) 48 (21.5%) 42 (22.8%)	0.838 0.877 0.505
Primary workplace location			
One of the five biggest cities Another smaller city Township/rural area	298 (79%) 97 (77.6%) 25 (80.6%)	79 (21%) 28 (22.4%) 6 (19.4%)	0.811
Workload (Full-Time equivalent)			
< 1 FTE 1 FTE > 1 FTE	16 (72.7%) 203 (75.5%) 201 (83.1%)	6 (27.3%) 66 (24.5%) 41 (16.9%)	0.086
Average work experience after finished training (years) *M* (*SD*) ^a^	20.65 (± 13.29)	18.34 (± 13.04)	0.107
Career change ideation			
Yes No	253 (73.1%) 167 (89.3%)	93 (26.9%) 20 (10.7%)	**< 0.001**
Poor working conditions			
Yes No	152 (72.7%) 268 (82.7%)	57 (27.3%) 56 (17.3%)	**0.006**
High workload			
Yes No	256 (79.5%) 164 (77.7%)	66 (20.5%) 47 (22.3%)	0.624
Work with patients			
Yes No	95 (76.6%) 325 (79.5%)	29 (23.4%) 84 (20.5%)	0.497
Lack of professional development			
Yes No	54 (69.2%) 366 (80.4%)	24 (30.8%) 19.6 (19.6%)	**0.025**
Lack of career perspectives			
Yes No	79 (66.9%) 341 (82.2%)	39 (33.1%) 74 (17.8%)	**< 0.001**
Managers			
Yes No	149 (69.6%) 257 (85.4%)	65 (30.4%) 44 (14.6%)	**< 0.001**
Mobbing			
Yes No	132 (68.4%) 288 (84.7%)	61 (31.6%) 52 (15.3%)	**< 0.001**
Exhaustion			
Yes No	270 (75.8%) 139 (84.8%)	86 (24.2%) 25 (15.2%)	**0.021**
Adequate salary			
Yes No	130 (79.8%) 290 (78.4%)	33 (20.2%) 80 (21.6%)	0.729
Satisfaction with work			
Yes No	213 (82.6%) 207 (75.3%)	45 (17.4%) 68 (24.7%)	**0.018**
Professional development			
Yes No	121 (90.3%) 316 (79.2%)	13 (9.7%) 83 (20.8%)	**0.041**
Patients’ gratitude			
Yes No	102 (76.1%) 318 (79.7%)	32 (23.9%) 81 (20.%)	0.387
Support from colleagues			
Yes No	264 (78.8%) 156 (78.8%)	71 (21.2%) 42 (21.2%)	0.996

Note. Chi-Square was used for all univariate tests, except if indicated - ^a^ Student t-test was used as a statistical model for comparison

^b^ responders could choose more than one response option

### Predictors of suicide risk

Multivariable binary logistic regression was conducted to evaluate the role of suicide risk factors, including sociodemographic characteristics, work-related stressors, depression and anxiety, and resilience. The entire model containing all predictors was statistically significant, χ^2^ [12] = 116.16, *P* < 0.001. The model explained between 20.4% (Cox and Snell R^2^) and 31.7% (Nagelkerke R^2^) of the variance in suicidality and correctly classified 81.1% of cases. Managers were a negative work-related factor with OR = 0.54 (*p* = 0.025), and extremely severe depression and anxiety were significant risk factors with OR of 3.8 and 7.6 (*p* < 0.001) for higher suicide risk, respectively. Lower resilience was an important predictor for high suicide risk OR = 0.97 (*p* = 0.027). Detailed analysis is presented in Table 6.

**Table 6:** Multivariate analysis of risk factors associated with high suicide risk among nurses

		95.0% CI for Odds Ratio		
Variable	Odds ratio	Lower	Upper	p
Not having kids	1.4	0.84	2.35	0.191
Career change ideation	1.3	0.69	2.48	0.413
Poor working conditions	1.26	0.72	2.22	0.415
Lack of professional development	0.74	0.38	1.45	0.384
Lack of career perspectives	0.73	0.39	1.35	0.318
Managers	0.54	0.31	0.92	**0.025**
Mobbing	0.74	0.44	1.26	0.364
Exhaustion	0.9	0.5	1.62	0.739
Satisfaction with work	1.09	0.66	1.81	0.735
Depression				
Normal* Mild Moderate Severe Extremely severe	1 1.02 1.71 2.3 3.79	- 0.4 0.78 0.88 1.26	- 2.58 3.75 6.05 11.36	- 0.963 0.182 0.091 **0.017**
Anxiety				
Normal* Mild Moderate Severe Extremely severe	1 2.79 3.6 2.26 7.61	- 0.88 1.46 0.79 2.77	- 8.78 8.87 6.44 20.85	**-** **0.071** **0.005** 0.127 **< 0.001**
Resilience	0.97	0.94	0.99	**0.027**

* - Normal value was used as a reference in a comparison

## Discussion

This study is the first attempt to evaluate the mental health among Lithuanian nurses in a national survey. We found out that 18% of nurses reported severe and extremally severe levels of depression symptoms, 29.3% - anxiety, and 17.1% stress. Around one-fifth of the sample (21.2%) had a high lifetime suicide risk, and 1.5% reported a previous suicide attempt.

A meta-analysis by Huang et al. [7] showed that among nurses in the intensive care unit, depression prevalence was almost 25%. Another meta-analysis [23] showed that 22% of nurses had depression. The data in our study was collected during the COVID-19 pandemic, which might have impacted the mental health of the sample. At the time of data collection in Lithuania, all healthcare employees were vaccinated against COVID-19, and more than half had booster vaccines. However, the pandemic was associated with a high workload and work-related stressors in healthcare systems. We found higher levels of depression in comparison to previous studies, with 18% of nurses having severe and highly severe depressive symptoms, and in addition to this, 29.8% had moderate depression symptoms. It is more than two times higher than the general population, with an average prevalence of 7.2% [24], according to epidemiological studies.

Anxiety and anxiety disorders are significant for healthcare workers. In our study, 32% of nurses had moderate anxiety symptoms, and 29.3% had severe and extremely severe anxiety symptoms. The prevalence of anxiety disorders ranged from 23.2 to 37% [23, 25, 26] based on several published metanalyses of healthcare staff in other studies. Prolonged mental problems can cause lower motivation, leading to poorer care for patients [27], major depression, risk of cardiac events, worsened quality of life and relationships [28], and lastly, severe anxiety can be associated with increased suicide risk [28, 29].

We identified that low resilience was associated with poor mental health, and lower resilience was significantly related to high suicide risk in logistic binary regression. Resilience is the ability to adapt to stress and adverse situations [30]. Yu et al. showed that stress, burnout, post-traumatic stress disorder and bullying were associated with poorer resilience [31]. A high-volume meta-analysis from Cochrane showed that resilience training might positively affect healthcare workers. However, the evidence for resilience training is uncertain [4]. Therefore, promoting mental health in nurses should focus on several directions: addressing work conditions, reducing work-related stressors, and providing resilience training.

Using the SBQ-R questionnaire, we identified that 21.2% of our study participants had a high suicide risk. Suicidal ideation differs from country to country and the specific populations, and it ranges among nurses from 5.2 to 62% [32–35]. High variability of suicide risk prevalence is associated with the methodology used in these studies. However, our study indicates worrying numbers of suicide risk among nursing staff in Lithuania. The binary logistic regression identified that depression and anxiety were significant risk factors increasing suicide risk up to 3.8 and 7.6 times. The findings are in line with previous studies, which revealed that depression and hopelessness can increase the death risk by suicide up to 1.9 to 2.2 times [36, 37]. In addition, anxiety is also a proven risk factor, contributing to increased suicide risk in previous studies [29, 37].

Coping with the COVID-19 pandemic puts additional strain on all healthcare workers’ psychological well-being and increases the burden of existing mental health problems [38]. In the early stages of the COVID-19 outbreak in Wuhan, frontline workers especially female nurses suffered the most due to depression, anxiety, insomnia and distress [39]. Additionally, Cai et al. [40] indicated that nurses experienced higher anxiety and nervousness relative to other healthcare professionals. Lastly, some reviews hypothesized that the COVID-19 pandemic can be an independent risk factor for worsened mental health [41]. However, we need not forget that the COVID-19 pandemic may not be the last; as shown in the 2015 MERS outbreak [42], frontline workers had the highest risk for post-traumatic stress disorder symptoms during the pandemic. Such outbreaks and pandemics will be a huge burden for all medical staff in the future, especially those who are closely involved with patients, especially nurses.

Our study had some limitations. First, this was a cross-sectional study, and we did not use a longitudinal design; therefore, future longitudinal studies could provide more information on the role of various risk factors on mental health changes in Lithuanian nurses. Second, while we aimed to conduct a large-scale national study of healthcare workers’ mental health, the recruitment of the study participants was not random and included self-referred participants willing to respond to our survey. Lastly, COVID-19 might have had an impact on the study findings. At the time of data collection, the COVID-19 pandemic was ongoing, and patients and healthcare workers were still exposed to its dangers. However, the data collection coincided with the third wave of the COVID-19 pandemic. There were enough protection measures in hospitals, the Lithuanian Ministry of Health had issued sufficient information and guidelines on how to deal with patients, and there was an experience of how to deal with patients because the country has already been living with the COVID-19 pandemic for more than one and a half years. In addition, all health workers (including nurses) were vaccinated on a priority basis and had already received 2 or more vaccines [43]. Due to these factors and the lack of sufficient baseline data, we are unable to determine the extent of the COVID pandemic’s impact on our nursing staff. Lastly, COVID-19 has not disappeared; revaccination is ongoing every year, and the virus has become a part of life in societies [44]. The mental health of the medical community has always been a huge concern. Since nurses and other medical practitioners play a vital role in society, it is essential to conduct periodic investigations and evaluations of this community to understand their challenges. Our investigation into this population revealed that nurses require improved mental health care to reduce depression and anxiety and prevent suicides. Secondly, our multivariate analysis showed that improving healthcare management could lead to mental health promotion in nurses. While significant structural changes in healthcare services and institutions are challenging and time-consuming, our study reveals that resilience training can enhance stress control, potentially reduce psychological distress, improve mental health, and potentially reduce suicidality. Finally, for mental health promotion in nurses, it is essential to target and reduce exhaustion, increase satisfaction with work, improve working conditions, reduce the workload, and, most importantly, eliminate mobbing.

## Conclusion

We evaluated a significant part of the Lithuanian nurse population to delineate mental health problems. We found that a substantial proportion of nurses in Lithuania had high levels of depression and anxiety problems. In addition to this, 21.2% had increased suicide risk. It is essential to address these problems in the national health care system to prevent the medical community and society from losing its valuable members and patients from losing essential caretakers.

## Data Availability

The datasets generated and/or analysed during the current study are not publicly available due sensitive origin but may be available from the corresponding author on reasonable request and with permission of Vilnius university, Faculty of Medicine.
